# Identification of the neuropeptide precursor genes potentially involved in the larval settlement in the Echiuran worm *Urechis unicinctus*

**DOI:** 10.1186/s12864-020-07312-4

**Published:** 2020-12-14

**Authors:** Xitan Hou, Zhenkui Qin, Maokai Wei, Zhong Fu, Ruonan Liu, Li Lu, Shumiao Bai, Yubin Ma, Zhifeng Zhang

**Affiliations:** 1 Ministry of Education Key Laboratory of Marine Genetics and Breeding, College of Marine Life Sciences, Ocean University of China, Qingdao, 266003 China; 2 Laboratory of Tropical Marine Germplasm Resources and Breeding Engineering, Sanya Oceanographic Institution, Ocean University of China, Sanya, 572000 China; 3 Hebei Research Institute of Marine and Fishery Science, Qinhuangdao, 066002 China; 4 College of Medical Engineering, Jining Medical University, Jining, 272067 China

**Keywords:** *Urechis unicinctus*, Echiura, Neuropeptide precursor, Larval settlement

## Abstract

**Background:**

In marine invertebrate life cycles, which often consist of planktonic larval and benthonic adult stages, settlement of the free-swimming larva to the sea floor in response to environmental cues is a key life cycle transition. Settlement is regulated by a specialized sensory–neurosecretory system, the larval apical organ. The neuroendocrine mechanisms through which the apical organ transduces environmental cues into behavioral responses during settlement are not fully understood yet.

**Results:**

In this study, a total of 54 neuropeptide precursors (pNPs) were identified in the *Urechis unicinctus* larva and adult transcriptome databases using local BLAST and NpSearch prediction, of which 10 pNPs belonging to the ancient eumetazoa, 24 pNPs belonging to the ancient bilaterian, 3 pNPs belonging to the ancient protostome, 9 pNPs exclusive in lophotrochozoa, 3 pNPs exclusive in annelid, and 5 pNPs only found in *U. unicinctus*. Furthermore, four pNPs (*MIP*, *FRWamide*, *FxFamide* and *FILamide*) which may be associated with the settlement and metamorphosis of *U. unicinctus* larvae were analysed by qRT-PCR. Whole-mount in situ hybridization results showed that all the four pNPs were expressed in the region of the apical organ of the larva, and the positive signals were also detected in the ciliary band and abdomen chaetae. We speculated that these pNPs may regulate the movement of larval cilia and chaeta by sensing external attachment signals.

**Conclusions:**

This study represents the first comprehensive identification of neuropeptides in Echiura, and would contribute to a complete understanding on the roles of various neuropeptides in larval settlement of most marine benthonic invertebrates.

## Background

Most marine benthic invertebrates have planktonic larvae during their life cycle. After going through a pelagic period, these planktonic larvae settle to the bottom and metamorphose into benthonic individuals (crawling, attaching, fixing and burrowing) [1, 2]. The larval settlement is the key event for their development and survival, which commonly includes the cessation of swimming and the appearance of substrate exploratory behavior [3–6]. This is a complex process determined by the interaction of biotic and abiotic factors at different temporal and spatial scale [7, 8]. The apical organ, a cluster of sensory neurons at the anterior of the larva in diverse groups as phoronids, polychaetes and chitons, has been implicated to be the site of perception cues for settlement and metamorphosis [9, 10]. Researchers found that neuropeptides expressed in chemosensory–neurosecretory cells of the apical organ can innervate ciliary bands, and suggested that they may play a role in the regulation of larval locomotion [11–14], which contribute to the larval settlement behavior [9].

Neuropeptides are considered to be the oldest neuronal signaling molecules in metazoans [15], and participate in the control of neural circuits and physiology [16–18]. They are generated from inactive precursor proteins by proteolytic cleavage and further modification such as C-terminal alpha-amidation and N-terminal pyroglutamination [19, 20], and then released into the hemolymph as hormones or the synapses as nerotransmitters to regulate the physiological activities of target cells [21]. Studies on the marine invertebrate larval neuropeptide systems have mainly been focused on lophotrochozoan including Mollusca and Annelida. For example, in mollusca, they mainly focus on larval development, larval feeding behavior, larval muscle innervation and muscular contractions [22–25]. In annelid *Platynereis*, neuropeptides have been indicated to involve in the ciliary beating, some neuropeptides (RYa, FVMa, DLa, FMRFa, FVa, LYa, YFa SPY and L11) for the larval upward swimming and others (FLa and WLD) for the downward swimming [21]. Furthermore, MIPs (myoinhibitory peptide) have been experimentally verified to play a role in regulating the larval settlement of marine annelid [9]. So far, other neuropeptides related to the larval settlement remain to be explored.

*Urechis unicinctus* is a representative species in Echirua inhabiting the U-shaped burrows in the coastal mud flats, and is also a commercial echiuran worm in China, Japan and Korea. The worm has a typical free-swimming trochophore larva beginning with the early trochophore stage (ET, 2 days post-fertilization; dpf) and the planktonic larva settles to the bottom during the segmentation larva stage (SL, 35 dpf; also called competent larva, CL), and then burrows the sediment and metamorphoses into the benthic worm (worm-shaped larva, WL, 42 dpf). Previous studies indicated that the SL stage larvae will delay metamorphosis and their mortality rate will increase if they do not find the adaptive substrate [3, 26, 27]. In this study, to provide a basic profile of the neuropeptide precursors for investigating the role of neuropeptides in *U. unicinctus* larval settlement, we screened the neuropeptide precursors potentially involved in the larval settlement from the *U. unicinctus* larval and adult transcriptomes. Furthermore, expression characteristics of the candidate genes were validated by qRT-PCR and whole-mount in situ hybridization. To map the candidate genes to the nerve cells at the special sites, nervous system in *U. unicinctus* larvae was analyzed using the fluorescence immunohistochemistry. The aim of this study was to identify neuropeptide precursors potentially involved in the larval settlement in the *U. unicinctus* and to provide new insights in larval settlement of marine benthic invertebrates.

## Results and discussion

### Overview of the neuropeptide precursors in *U. unicinctus*

**Fig. 1:**
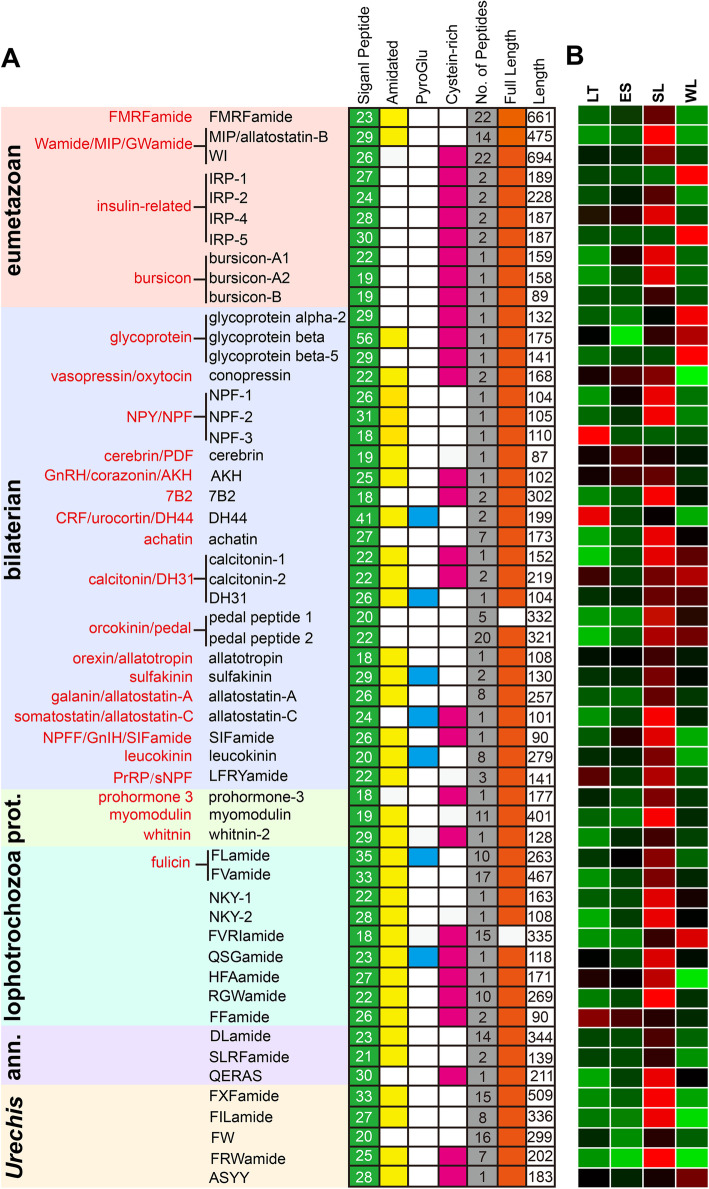
Summary of the identified neuropeptide precursors from *U. unicinctus* larval and adult transcriptomes. **a** pNPs are classified based on their phylogenetic distribution into eumetazoan, bilaterian, protostome (prot.), lophotrochozoan, annelid (ann.) and *Urechis*-specific. Previously established metazoan neuropeptide families are indicated in red [28]. **b** Hierarchical clustering of the neuropeptide precursor genes in *U. unicinctus* larval transcriptomes [29]. LT, late-trochophore (25 dpf, pelagic larva); ES, early- segmented larva (32 dpf, pelagic larva); SL, segmented larva (35 dpf, competent larva); WL, worm-shaped larva (42 dpf, benthic larva). Colors represent the gene expression levels from green (low), black (middle) to red (high)

We performed BLAST search and NpSearch prediction to screen the neuropeptide precursors in the transcriptomes of *U. unicinctus*. A total of 54 neuropeptide precursors (pNPs) were identified, 7 from BLAST search, 5 from NpSearch prediction, and 42 from both methodologies (Fig. 1a and Supplementary Table S2). Among them, 49 pNPs had been reported in other species, and the remaining 5 pNPs were first identified in *U. unicinctus* and we named them FxFamide, FILamide, FW, FRWamide and ASYY according to their conserved amino acid residues. In the *U. unicinctus* transcriptomes, most neuropeptide precursor sequences contained the full-length open reading frame (ORF) with a signal peptide (SP), except pedal peptide 1 and FVRIamide. The sequence characteristics of *U. unicinctus* neuropeptide precursors for the SP presence, the conserved peptide motifs and other hallmarks of bioactive peptides, e.g. amidation C-terminal Gly, Cys-containing stretches, mono- or dibasic cleavage sites were summarized in Fig. 1a and Supplementary Fig. S1.

Due to the inherent difficulties of analyzing highly diverse and repetitive pNPs, the relationships among different families are often elusive. Therefore, Jékely [28] and Conzelmann [30], using similarity-based clustering and sensitive similarity searches, obtained a global view of metazoan pNP diversity and evolution based on a curated dataset of 6225 pNPs from 10 phyla. This approach was also useful for analyzing the phylogenetic distribution of *U. unicinctus* pNPs and we classified the pNP families using the same methodology. The results showed that ten pNPs in *U. unicinctus* were categorized as the ancient eumetazoan families (Fig. 1a), which are the repertoire neuropeptides with the short amidated peptides, such as R [F/Y] amide, Wamide, insulin-related peptide and the glycoprotein hormones [28, 31, 32]. Then, twenty-four pNPs in *U. unicinctus* were categorized as the ancient bilaterian families (Fig. 1a), which belong to 17 neuropeptide families [28]. Three members of the ancient protostome neuropeptide precursor families were present in *U. unicinctus*, including prohormone-3, myomodulin, and whitnin-2 (Fig. 1a). Moreover, we identified nine pNPs in *U. unicinctus* (Fig. 1a), which were proposed to be the lophotrochozoan-specific families [30]. Three pNPs in *U. unicinctus* had recognizable orthologs only in annelids, including DLamide, SLRFamide and QERAS (Fig. 1a). In addition, five pNPs did not have recognizable orthologs outside *Urechis*, and were temporarily classified as neuropeptides unique to *U. unicinctus*, including the FxFamide, FILamide, FW, FRWamide and ASYY (Fig. 1a).

Traditionally Echiura was ranked as a phylum, but recent studies, especially on molecular phylogenetic analysis [33] and morphological observation [34, 35], have generated an increasing body of evidence that they actually are derived annelids and provide strong support for a sister group relationship between Echiura and Capitellidae. This is consistent with our study in which we find three Annelid-specific pNPs were presented in *U. unicinctus*.

### Screen of the neuropeptide precursors potentially involving in the larval settlement

We performed a hierarchical clustering of the neuropeptide precursors based on their stage-specific expression (FPKM values) from the *U. unicinctus* transcriptomes (Fig. 1b and Supplementary Fig. S2). The results showed that most of the neuropeptide precursors were expressed at multiple stages, and the expression levels were significantly different. We found when the larvae developed from LT to ES, a process which the larvae initially transited from upper to middle layer in the water column, expression levels of *NPF-3* and *DH44* decreased significantly (*p* < 0.05), while that of *bursicon-A2* and *NPF-1* was significantly increased (*p* < 0.05) (Fig. 1b and Supplementary Fig. S2). During the development progress from ES to SL, a period that the larvae move from the middle to the bottom of water layer, eleven pNP genes (*MIP*, *bursicon-A2*, *NPF-2*, *RGWamide*, *7B2*, *pedal peptide 1*, *myomodulin*, *FVRIamide*, *FxFamide*, *FILamide* and *FRWamide*) were significantly up-regulated (*p* < 0.05) (Fig. 1b and Supplementary Fig. S2). However, eight genes (except *7B2*, *pedal peptide 1* and *FVRIamide*) among the eleven pNPs above were again down-regulated (*p* < 0.05) when the larvae developed from SL to WL, which is the period that the larvae begin to explore the suitable substrate and finally became benthic larvae (Fig. 1b and Supplementary Fig. S2). As *MIP* have been confirmed to regulate larvae settlement behavior [9], we speculated preliminarily the eight pNPs with similar expression pattern were considered to be most likely pNPs involved in the regulation of larval settlement and metamorphosis in *U. unicinctus*.

### Sequence characteristics of the selected pNPs that may be involved in the regulation of larval settlement in *U. unicinctus*

Four interesting pNPs, including previously reported MIP [9] and three *Uu-specific* pNPs (FxFamide, FILamide and FRWamide), were selected for further analysis.

**Fig. 2:**
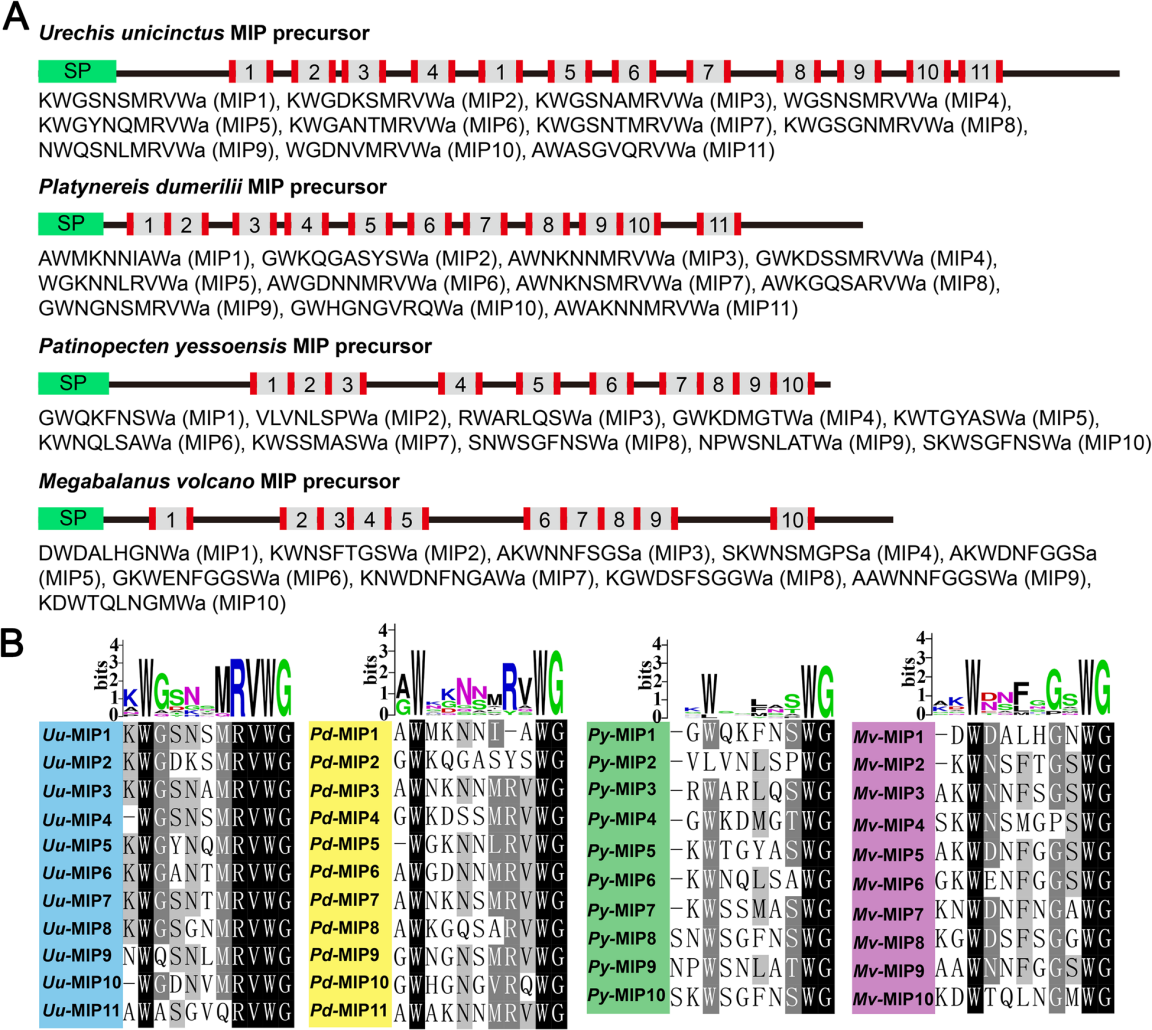
Schematics of MIP precursors and alignment of potential bioactive peptides. **a** Schematics of MIP precursor proteins for the echiuran *Urechis unicinctus* (GenBank: MT162087), annelid *Platynereis dumerilii* (GenBank: JX513877), mollusc *Patinopecten yessoensis* (GenBank: MH045202) and arthropod *Megabalanus volcano* (GenBank: MF579246). N-terminal signal peptides are showed in green, the predicted peptides in gray and the basic cleavage sites on the flanked of the predicted peptides in red. The serial number in each MIP precursor represents the types of the predicted mature MIPs. **b** Multiple alignments and peptide logos of the predicted mature MIPs from *U. unicinctus* (*Uu*), *P. dumerilii* (*Pd*), *P. yessoensis* (*Py*) and *M. volcano* (*Mv*)

MIPs (Myoinhibitory peptides) are pleiotropic neuropeptides first described in insects as inhibitors of muscle contractions [18, 36, 37]. In some insect species, MIPs modulate juvenile hormone synthesis and reduce food intake, and they are also referred to as allatostatin-B or WWamide [38–41]. In *Platynereis* the MIPs have been confirmed to regulate larvae settlement behavior [9] and feeding behavior [42]. They are characterized by a conserved domain containing two Trp residues which are usually separated by five to eight amino acid residues in insects, molluscs and annelids [43–45]. In *U. unicinctus* transcriptomes, we identified a neuropeptide precursor which is an orthologue of arthropod MIP (Fig. 2). The *Uu-*MIP precursor contains 11 mature peptides, the number of mature peptides in *U. unicinctus* is the same as that of the annelid *Platynereis dumerilii*, while differs from the mollusc *Patinopecten yessoensis* and the arthropod *Megabalanus volcano* which have 10 mature peptides (Fig. 2a). Sequence alignment of the bioactive peptides revealed that the sequence similarity among the different mature MIPs in *U. unicinctus* was higher than that in *P. dumerilii*, *P. yessoensis* and *M. volcano* (Fig. 2b). Moreover, the MRVWamide motif in C-terminal of the mature MIPs is present in *U. unicinctus* and *P. dumerilii*, but not in *P. yessoensis* and *M. volcano* (Fig. 2b). The above results show that the characteristics of the MIP precursor sequence of *U. unicinctus* are closer to that of *P. dumerilii*, which is consistent with the classic species evolution.

**Fig. 3:**
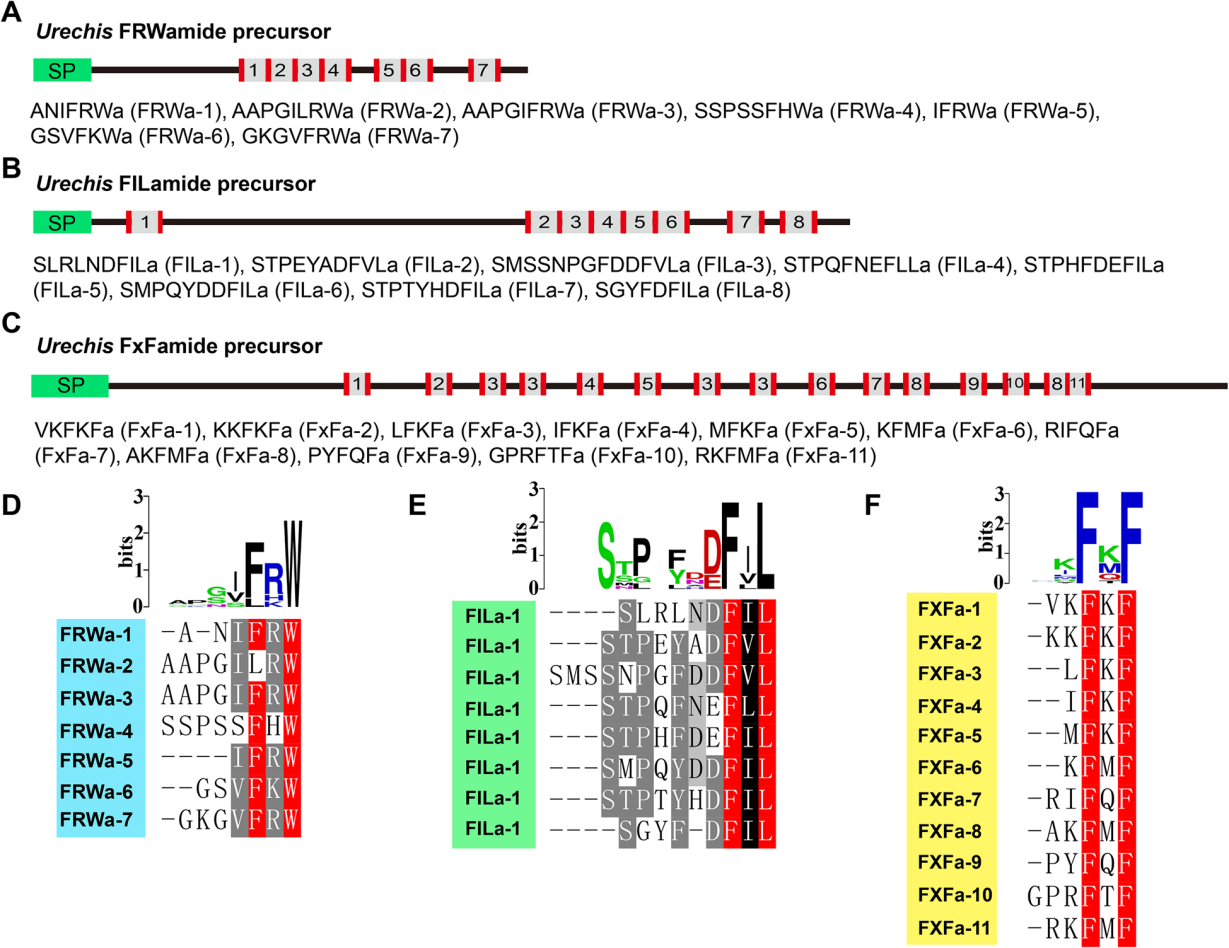
FRWamide, FILamide and FxFamide neuropeptide precursors. **a**, **b** and **c** Schematics of FRWamide, FILamide and FxFamide precursor proteins in *U. unicinctus*. N-terminal signal peptides (green) and the predicted peptides (gray) flanked by basic cleavage sites (red) are shown. The predicted mature FRWamide, FILamide and FxFamide sequences and their numbering as used in the text are listed. **d**, **e** and **f** Multiple alignments and peptide logos of the predicted mature FRWamide (GenBank: MT162138), FILamide (GenBank: MT162136) and FxFamide (GenBank: MT162135)

In this study, five potential neuropeptide precursors were for the first time identified in *U. unicinctus* (Fig. 1a), and three of them (FRWamide, FILamide and FxFamide) were predicted to play a role in regulating *U. unicinctus* larvae settlement based on the significant differences in mRNA level from the segmented larvae to worm-shaped larvae (Fig. 1b). FRWamide precursor is comprised of 202 amino acids which contains a 25-residue signal peptide and 7 copies of neuropeptides with FRWamide motif in the C-terminal (Fig. 3a and d). FILamide precursor is comprised of 336 amino acids which contains a 27-residue signal peptide and 8 copies of neuropeptides with FILamide motif in the C-terminal (Fig. 3b and e). FxFamide precursor is comprised of 509 amino acids which contains a 33-residue signal peptide and 15 copies of neuropeptides with FxFamide motif in the C-terminal (Fig. 3c and f). These newly discovered neuropeptide precursors enrich the intension of neuropeptide composition.

### Spatio-temporal expression of the selected pNPs during the larval settlement

**Fig. 4:**
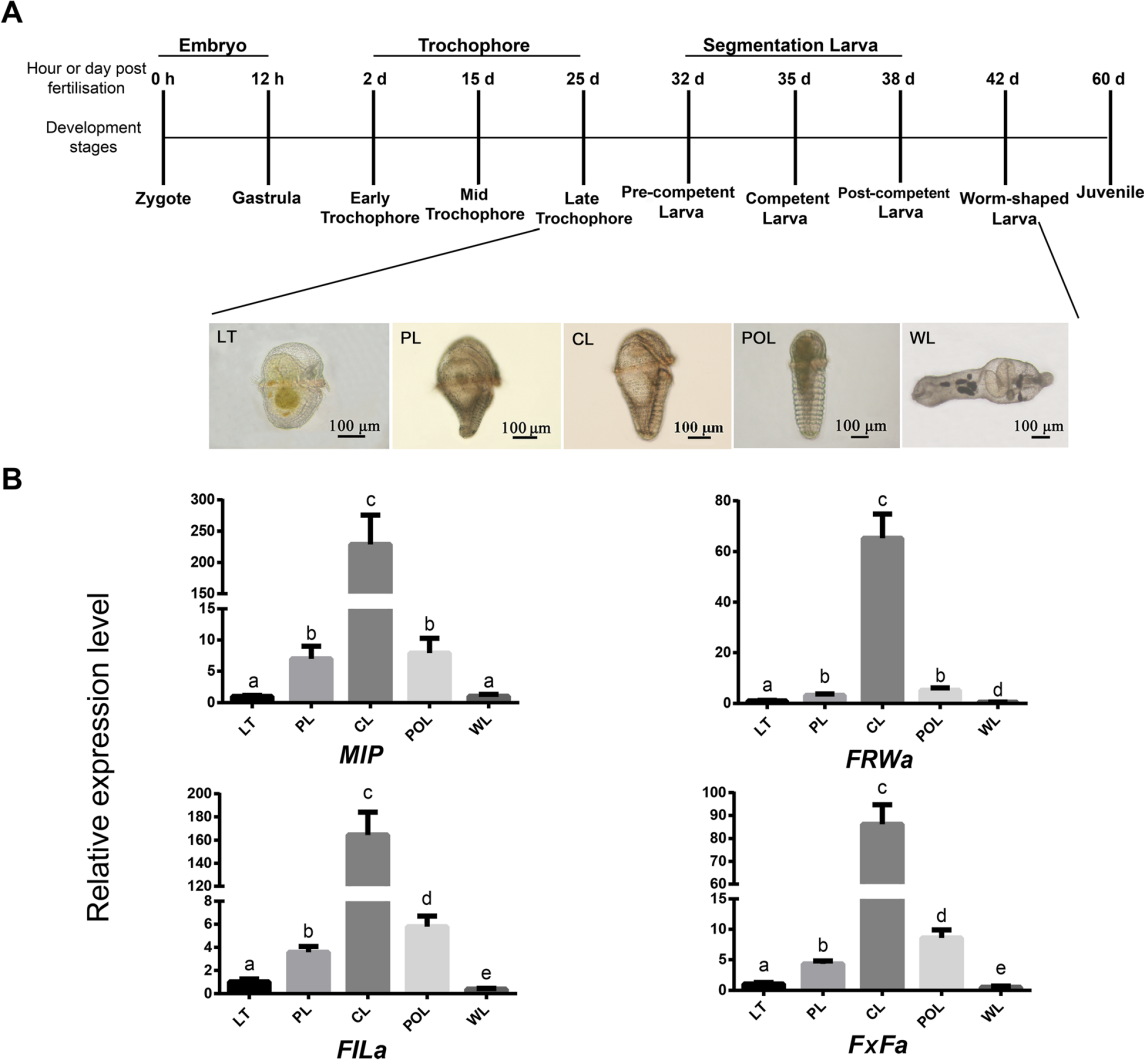
The relative expression levels of the pNP genes in *U. unicinctus* larvae during the settlement. **a**, A time course of *U. unicinctus* development indicating qRT-PCR sampling strategy employed in this study. **b**, the relative expression levels of the pNP genes. LT, late-trochophore (25 dpf, pelagic larva); PL, pre-competent larva (32 dpf, correspond to ES in transcriptome data); CL, competent larva (35 dpf, correspond to SL in transcriptome data); POL, post-competent larva (38 dpf); WL, worm-shaped larva (42 dpf, benthic larva). Data are indicated as mean ± SD from triplicate experiments and analyzed using One-way ANOVA followed by Tukey’s HSD test. Different letters indicate significant difference between different developmental stages (*p* < 0.05)

To verify the expression of the four pNP transcripts (*MIP*, *FILamide*, *FxFamide* and *FRWamide*), *U. unicinctus* larvae including late-trochophore (LT), pre-competent larva (PL), competent larva (CL), post-competent larva (POL) and worm-shaped larva (WL) were employed for qRT-PCR analysis (Fig. 4a). The results showed that the mRNA levels of the four pNP genes increased through larval development, with the highest expression in CL, and then significant decrease in POL and WL (Fig. 4b). These results are consistent with the transcriptome data (Fig. 1b and Supplementary Fig. S2). During the developmental progression from LT to CL, the *U. unicinctus* larvae move from the upper to the middle layer in water, and gradually acquire the ability to explore a suitable substrate in CL, finally become benthic larvae in WL. Thus, we suggested that these four genes may be involved in the biological activities of the larvae exploring the substrate for settlement in *U. unicinctus*.

**Fig. 5:**
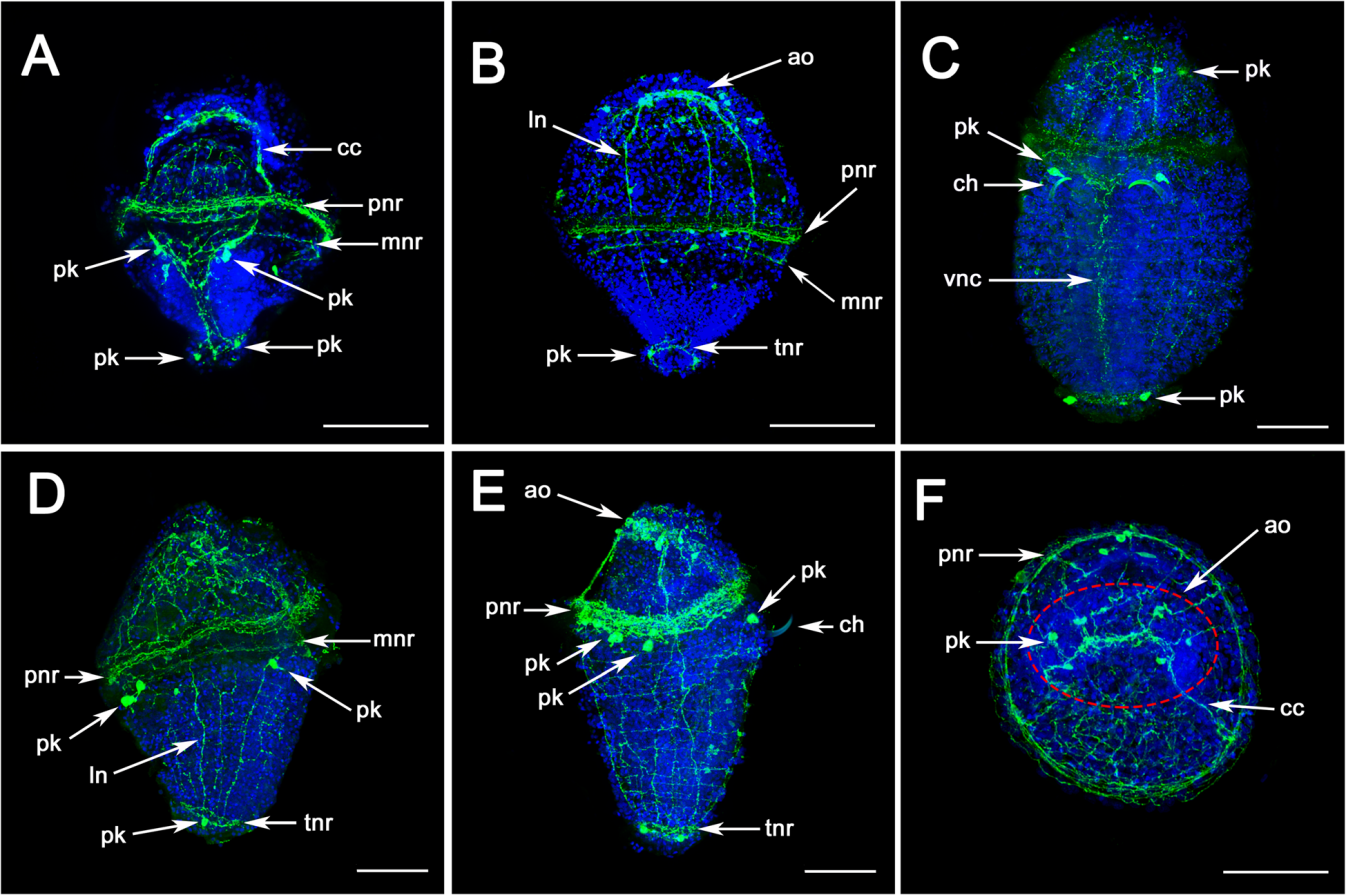
The nervous system in *U. unicinctus* larvae detected by Immunofluorescence technique with 5-HT antibody. **a-b** correspond to late-trochophore (LT) of *U. unicinctus*. **c-f** correspond to competent larva (CL); **a** and **c**, ventral view; **b** and **e**, dorsal view; **d**, lateral view; **f**, anterior view of CL. ao, apical organ; cc, circumoesophageal connectives; ch, chaeta; ln, longitudinal nerve fibre; mnr, metatroch nerve ring; pk, perikarya; pnr, prototroch nerve ring; tnr, telotroch nerve ring; vnc, ventral nerve cord. Scale bars: 200 μm

To map the expression of these pNPs (*MIP*, *FxFamide*, *FILamide* and *FRWamide*) to nerve cells at the special sites, nervous system in *U. unicinctus* larvae was analyzed using fluorescence immunohistochemistry with an anti-5HT antibody (Fig. 5). The results showed that, in trochophore up to an age of approximately 15 days, only a few structures of the nervous system are labeled with antibodies against 5-HT. In the episphere of the larvae, the circumoesophageal connectives (CC) and two nerve rings innervating the prototroch and metatroch are visible (Fig. 5a). In the hyposphere of the larva, two longitudinal nerves (LN) merge after a short distance forming a median nerve named ventral nerve cord (VNC). Two pairs of perikarya are discernible in the anterior region, directly behind the slit-shaped mouth opening (Fig. 5a, c) and on the telotroch nerve ring (Fig. 5a, b, c). In dorsal view of the larva, 3–4 LNs can be seen in the episphere which connect to the prototroch and metatroch nerve rings (Fig. 5b). As development proceeds up to the competent larva, in which the anterior chaetae have already been formed, the paired longitudinal nerve tracts of the VNC are fused in the ventral midline (Fig. 5c). In addition, the metatroch nerve ring is disappeared and two labeled perikaryas are visible on the dorsal side of the larva just under the prototroch nerve ring (Fig. 5d, e). The apical organ of the larva is shown in Fig. 5f. Fluorescence immunohistochemistry were also used to study the development of the nervous system in various other Echiuran species, such as *Bonellia viridis* [46, 47] and *Urechis caupo* [34]. Our results are consistent with those previous studies which have proven to be informative in the study of neurogenesis in neuronal structures of Echiurans. Besides, we revealed several previously unreported details — eight nerve fibers and six large labeled perikaryas are visible in the apical organ in Echiuran worm (Fig. 5f), which is similar to that reported in *P. dumerilii* [9, 21, 30, 48] and especially in *Capitella teleta* [48].

**Fig. 6:**
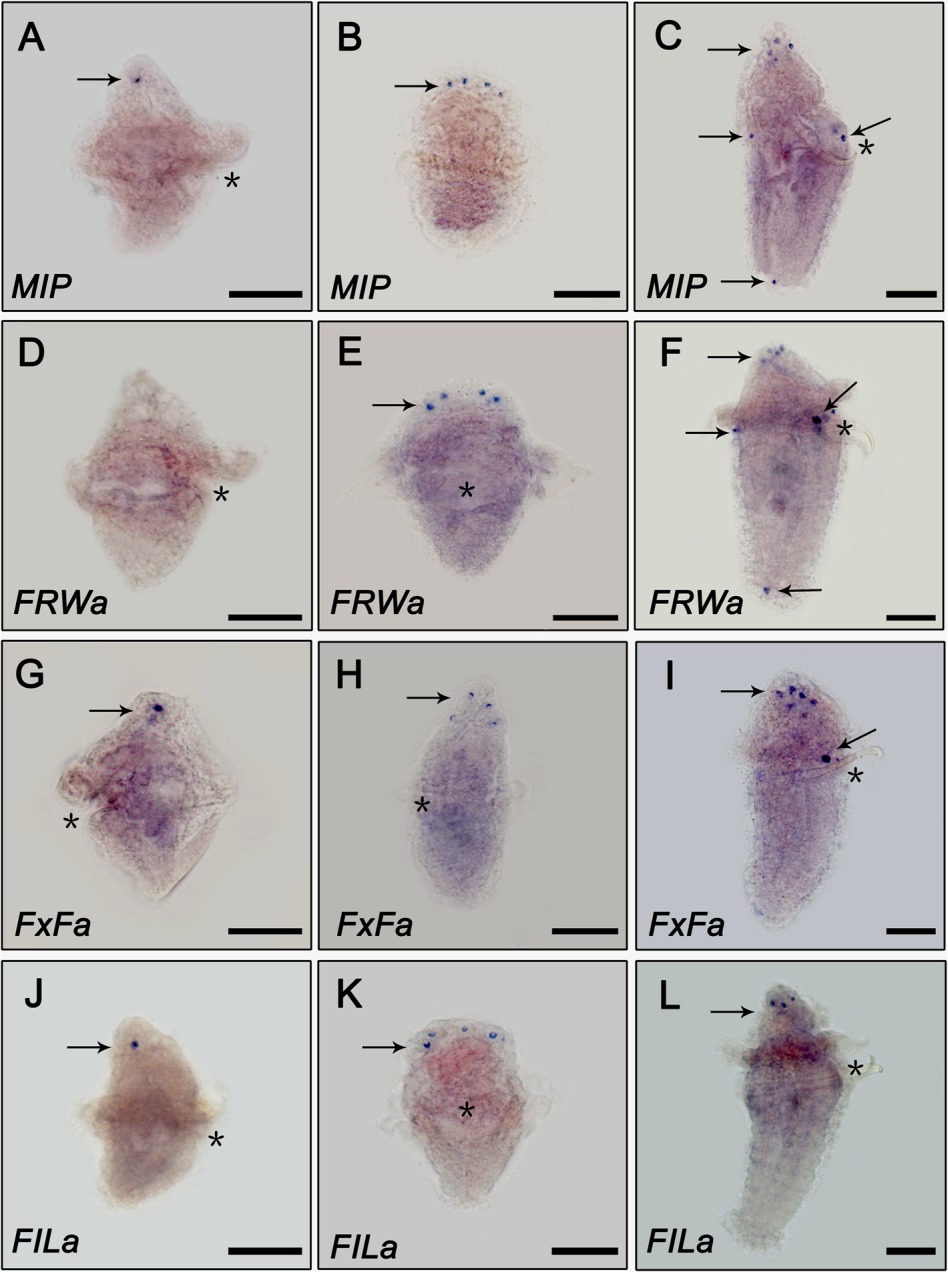
The expression patterns of *MIP*, *FRWa*, *FxFa* and *FILa* in *U. unicinctus* larvae detected by whole-mount in situ hybridization. **a**, **d**, **g** and **j** correspond to early-trochophore (2 dpf, pelagic larva); **b**, **e**, **h** and **k** correspond to late-trochophore (25 dpf, correspond to LT in transcriptome data, pelagic larva) and (**c**, **f**, **i** and **l**) correspond to competent larva (35 dpf, correspond to SL in transcriptome data). The asterisk indicates the location of the larvae mouth; **b**, dorsal view; **e** and **k**, ventral view; the remaining panels are all lateral views. Scale bars: 200 μm. Negative controls with sense probe can be found in Supplementary Figure S3

Next, location of four pNP mRNAs including *MIP*, *FxFamide*, *FILamide* and *FRWamide* were detected by Whole-mount mRNA in situ hybridization (WISH) (Fig. 6 and Figure S3). The results showed that a positive *MIP* signal was first observed in the central region of the episphere in the early-trochophore larva (Fig. 6a) which is similar to that of the apical organ in *C. teleta* and *P. dumerilii* [9, 48]. As the development proceeds, four positive cells are exclusively located in the apical organ of the late-trochophore larva (Fig. 6b). Until the competent larvae, the obvious positive signals were located in four regions, including the apical organ (the 4–6 cells), above the abdomen chaetae (the two cells), the prototroch in the dorsal side of the larvae (the two cells) and the both side of the telotroch (the two cells) (Fig. 6c). The expression patterns of *FRWamide*, *FxFamide* and *FILamide* were similar to that of *MIP* (Fig. 6), except no visible *FRWamide* positive signal was observed in *U. unicinctus* early-trochophore (Fig. 6d), in competent larvae *FxFamide* was detected only in apical organ (the six stained cells) and above the abdomen chaetae (the two stained cells) (Fig. 6i), while the positive signal of *FILamide* in the competent larva was only in the four positive cells of apical organ (Fig. 6l). Since the WISH experiment was observed after being sealed with resin, it was difficult to observe the apical view of the larva.

In many marine invertebrates the transition from free-swimming larvae to bottom-dwelling juveniles is regulated by neuroendocrine signals (including neuropeptides and hormones) [49–51]. In diverse ciliated marine larvae, the apical organ, has been implicated in the detection of cues for the initiation of larval settlement [9, 10, 52–55]. Previous studies suggest that several neuropeptides expressed in distinct sensory neurons (apical organ) innervate locomotor cilia, which contribute to the larvae swimming depth [9, 21]. The alternation of active upward swimming and passive sinking, together with swimming speed and sinking rate, is thought to determine vertical distribution in the water [56]. In *Platynereis*, neuropeptides including RYa, FVMa, DLa, FMRFa, FVa, LYa, YFa, FLa, MIP, GWa et al. can alter ciliary beat frequency and the rate of calcium-evoked ciliary arrests [9, 21], which eventually may be involved in regulating the larvae settlement. In our study, the *MIP* and *FRWamide* were detected in the dorsal side of the prototroch nerve ring and the both side of the telotroch nerve ring (Fig. 6c, f), indicating that they may play a role in *U. unicinctus* ciliary beating and eventually cause the larvae sinking to the bottom. MIP is the only neuropeptide that has been shown to be involved in larval settlement in *Platynereis* [9], which is expressed in chemosensory–neurosecretory cells in the annelid larval apical organ. The researchers found that synthetic MIPs can induce the settlement of *P. dumerilii* larvae, and they demonstrate by morpholino-mediated knockdown that MIP signals via a G protein-coupled receptor to trigger settlement [9]. These results reveal a role for a conserved MIP receptor–ligand pair in regulating marine annelid settlement. In this study, we revealed that *MIP*, *FxFamide*, *FILamide* and *FRWamide* all localized in the region of the apical organ (Fig. 6), like MIPs expression pattern in *P. dumerilii*, indicating that these neuropeptides may also be involved in triggering larval settlement. In addition, the expressions of the *MIP*, *FRWamide* and *FxFamide* were also detected at base of the abdomen chaetae (Fig. 6c, f and i). The chaetae are important in locomotion, stabilization during peristalsis, and sensing the environment in annelids [57], and have been implicated in assisting movement and stabilizing body segments within the tube for worms living in burrows or tubes [58, 59]. However, in sediment-dwellers they contact the inside walls of tubes or burrows. The cantilever nature of capillary chaetae and their astounding breadth of flexural stiffness suggest that they could be very effective at transmitting specific mechanical information about their surroundings to the body of the worm [34, 57]. Therefore, we propose that *MIP*, *FRWamide* and *FxFamide* located in base of the abdomen chaetae may help *U. unicinctus* larvae crawl on the sediment surface or explore the bottom and eventually contribute to larval settlement. Meanwhile, there are also some limitations to this study which need to be acknowledged. Firstly, this study only uses transcriptomic data to identify neuropeptides, therefore these predicted pNPs have not been confirmed by mass spectrometry to show that they are definitely released as signaling peptides in the worm. Secondly, we only used WISH technology to explore the location of the pNP genes at the mRNA level. Therefore, some issues including mass spectrometry, immunohistochemistry, western blot and other functional studies remained to be investigated in the future.

## Conclusions

In this study, we identified 54 pNP genes in *U. unicinctus* larvae and adult transcriptome databases based on BLAST and NpSearch prediction, and suggested that the neuropeptide system of *U. unicinctus* is very close to that of annelids according to their phylogenetic distribution. Based on the expression data of pNP genes in the transcriptome of *U. unicinctus* larvae, four pNPs that may be associated with larval settlement were selected to further investigation. qRT-PCR results showed that the four pNPs were indeed highly expressed in competent larvae. WISH results indicated all the four pNPs were expressed in the region of the apical organ of the larva, and the positive signals were also detected in the ciliary bands and abdomen chaetae. These results imply that the four pNPs may be involved in sensing settlement signals and regulating larval ciliary locomotor and the movement of chaeta, and eventually may play an important role in the settlement of *U. unicinctus* larvae. Our findings provide some basic data for investigate the complex regulatory mechanisms in larval settlement of marine benthic invertebrates.

## Methods

### Animals and sampling

Adult *U. unicinctus* were obtained from Jiutian aquatic products market in Zhifu District of Yantai, China. Sperm and ova were obtained by dissecting the nephridia (gonaduct) of male and female, respectively. Artificial insemination was conducted through mixing the sperm and ova with a ratio of 10: 1 in filtered sea water (FSW). The fertilized eggs were reared in FSW (17 °C, pH 7.7, and salinity 30 PSU), and the hatched larvae were fed with single-cell algae (*Isochrysis galbana*, *Chlorella vulgaris* and *Chaetoceros muelleri*). The larvae at different stages were sampled and fixed in 4% paraformaldehyde for 15 h at 4 °C, and then dehydrated with serial methanol (25, 50, 75 and 100%) and stored in 100% methanol at − 30 °C for immunofluorescent histochemistry and whole-mount in situ hybridization analysis. The larvae from five developmental stages, late-trochophore (LT, 25 dpf), pre-competent larva (PL, 32 dpf) correspond to early-segmented larva (ES), competent larva (CL, 35 dpf) correspond to segmented larva (SL) which is the fully developed larvae prior to settlement and metamorphosis [26], post-competent larva (POL, 38 dpf) correspond to late-segmented larva (LS), and worm-shaped larva (WL, 42 dpf) were collected (Fig. 4a), frozen with liquid nitrogen immediately and then stored at − 80 °C, respectively for total RNA extraction. Three biological replicates from each developmental stage were prepared.

### Identification, classification and sequence alignment of neuropeptide precursors

Data from *U. unicinctus* larval transcriptomes [29, 60] and adult transcriptome [61] were used in this study. To search for transcripts encoding putative neuropeptides or peptide hormone precursor proteins in *U. unicinctus*, the homologous sequences previously identified in annelids (*Platynereis dumerilii* [30], *Capitella capitate* [44] and *Helobdella robusta* [44]), molluscs (*Patinopecten yessoensis* [45], *Pinctata fucata* [62], *Lottia gigantea* [63], *Crassostrea gigas* [62] and *Deroceras reticulatum* [64]) and echinoderms (*Asterias rubens* [65], *Ophionotus Victoria* [66], *Strongylocentrotus purpuratus* [67] and *Apostichopus japonicus* [68]) were downloaded from NCBI and used as queries in tBLASTn searches of the assembled *U. unicinctus* transcriptome database using BioEdit v7.0.9 with an E-value cutoff of 1e-5. Open reading frames (ORFs) in these mRNA sequences of potential neuropeptides were identified using DNASTAR v7.1. The resultant protein sequences were further evaluated based on (i) the presence of a putative N-terminal signal peptide identified by SignalP 4.1 and Signal-3 L 2.0 [69, 70]; (ii) the presence of putative monobasic or dibasic cleavage sites flanking the putative bioactive peptides according to the existing neuropeptide cleavage motifs [71]; (iii) the presence of a C-terminal glycine residue which is a putative amidation site, and (iv) the presence of cysteine residues which can form disulfide linkages.

Furthermore, we used a neuropeptide-prediction tool NpSearch (https://github.com/wurmlab/npsearch) to identify the putative neuropeptide precursors with low sequence similarity to known precursors based on various characteristics (signal peptide, cleavage sites, C-terminal glycine and cysteine residues). Functional annotation of the identified neuropeptide precursors was finally conducted by searching against NCBI non-redundant protein sequence (nr) database using BLASTx algorithm with the E-value of 1e-5.

The classification of *U. unicinctus* neuropeptides is mainly based on the researches of Jékely [28] and Conzelmann [30], and we have also updated the classification status of LFRYamide [72, 73], Cerebrin [28, 63, 74, 75] and RGWamide [30, 44, 45, 62–64, 76, 77] according to recent studies.

The neuropeptide precursor homologous sequences from other species were collected from GenBank. Multiple alignments were conducted using ClustalW [78], and the results were annotated with GeneDoc (https://genedoc.software.informer.com). The frequency of each amino acid in the alignment result was presented using the online tool WebLogo [79]. The hierarchical clustering of the pNP genes according to their FPKM values in the *U. unicinctus* larval transcriptome [29] was performed by an online tool (https://www.omicshare.com/tools/).

### RNA isolation, cDNA synthesis and quantitative real-time PCR (qRT-PCR)

Total RNA from each stored larval sample was isolated using MicroElute®Total RNA Kit (Omega, Norcross, USA) according to the manufacture’s instruction. The cDNA was synthesized for each sample using Prime-Script™ RT reagent Kit (TaKaRa, Dalian, China) with the gene specific primers (Supplementary Table S1) designed using Primer Premier 5.0 according to their predicted CDS sequences. The amplifications were performed with SYBR Premix Ex Taq kit (TaKaRa, Dalian, China) in LightCycler 480 Real-Time PCR system. The PCR mixture consisted of 10 μl SYBR Premix Ex Taq II, 1 μl template cDNA, 1 μl forward primer (10 μM), 1 μl reverse primer (10 μM) and 7 μl ddH_2_O. The qRT-PCR condition was: denature at 95 °C for 30 s, followed by 39 cycles of 5 s at 95 °C, and 60 °C for 30 s. Each sample was run in 3 technical replicates. The relative expression levels were normalized to the reference gene *ATPase* [80], and expression ratios were calculated using the 2^–ΔΔCt^ method. The experimental data were presented as mean ± standard deviation from three samples with three parallel repetitions, and all RT-PCR assays were validated in compliance with “the MIQE guidelines” [81]. Significant differences between means were tested using one-way analysis of variance (ANOVA) followed by Tukey’s HSD test with SPSS software 18.0 (SPSS Inc., Chicago, USA). The significance level was set at *p* < 0.05.

### Whole-mount in situ hybridization (WISH)

Specific fragments from cDNA of each neuropeptide genes were amplified using the primers with T7 or Sp6 promoter sequence at their 5′ ends (Supplementary Table S1). DIG-labeled RNA probes were prepared using the DIG RNA Labeling Kit SP6/T7 (Roche, Basel, Switzerland) with the PCR products as templates. WISH was carried out with the following protocols: the larvae were rehydrated with PBT (PBS + 0.1% Tween-20), and treated with 200 ng/μl proteinase K for 30 min to optimize hybridization; pre-hybridization was carried out for 6 h at 60 °C, then the probe was added and incubated at last 16 h at 60 °C; after the excess probe was removed by several rinses in hybridization buffer (50% formamide, 5 × SSC, 0.1% Tween, 9.2 mM citric acid for adjustment to pH 6.0, 50 μg/mL heparin, 500 μg/mL yeast RNA), the non-specific binding sites in the larval cells were blocked using the blocking buffer; the samples were incubated with the Anti-DIG-AP antibody (Roche, Basel, Switzerland) for 16 h at 4 °C; finally, the samples were stained in an NBT/BCIP staining solution (Roche, Basel, Switzerland) and kept in the dark for 1 h, and then dehydrated. The results were observed and photographed using a Nikon E80i microscope (Nikon, Tokyo, Japan). Drawings and final panels were designed using Adobe Photoshop (Adobe, San Jose, CA, USA).

### Immunofluorescence histochemistry

The larva samples were rehydrated in a gradient methanol series (100, 75, 50 and 25%), and treated with 3% bovine serum albumin (BSA) (Shanghai biotechnology Technology, Shanghai, China) diluted by PBT (pH 7.4). Subsequently, the samples were transferred into primary antibody (Anti-5-hydroxytryptamine, an antibody produced in rabbit, Sigma, Jaffrey, USA) diluted 1: 200 in BSA and incubated overnight at 4 °C on a nutator. Afterwards, the samples were rinsed in PBT for 2 h and incubated subsequently with secondary fluorochrome conjugated antibodies (donkey anti-rabbit Alexa Fluor 488, Invitrogen, CA, USA) diluted 1: 300 in PBT for 2 h. At last, the larvae were washed six times in PBT and incubated in PBT with 2.5% DAPI (Solarbio, Beijing, China) in the dark for 15 min to label cell nuclei. Negative controls were obtained by pre-immune serum in order to check for antibody specificity. All the samples were analyzed with the confocal laser-scanning microscope Nikon A1RSi (Nikon, Tokyo, Japan). Drawings and final panels were designed using Adobe Photoshop (Adobe, San Jose, CA, USA).

## Supplementary Information

**Additional file 1: Table S1**. Specific primers used in this research.

**Additional file 2: Table S2**. Detailed information of the identified neuropeptide precursors in *U. unicinctus.*

**Additional file 3: Figure S1.** Structures of *U. unicinctus* pNPs and identified repetitive peptide motifs.

**Additional file 4: Figure S2.** Expression trends of the neuropeptide precursor genes in *U. unicinctus* larval transcriptome.

**Additional file 5: Figure S3.** The negative controls of *MIP*, *FRWa*, *FxFa* and *FILa* in *U. unicinctus* larvae detected by whole-mount in situ hybridization.

## Data Availability

The datasets analysed during the current study are available in the NCBI SRA repository (SRX397931, SRX398497, SRX4526076, SRX4526077, SRX4526078, SRX2999430, SRX4526079, SRX4526072, SRX4526073, SRX4526074, SRX4526075, SRX4526080, SRX2999431, SRX4526070, SRX4526071, SRX4526081).

## Abbreviations

pNPs: Neuropeptide precursors
qRT-PCR: Quantitative real-time PCR
ORF: Open reading frame
SP: Signal peptide
LT: Late-trochophore
ES: Early- segmented larva
SL: Segmented larva
WL: Worm-shaped larva
MIPs: Myoinhibitory peptides
PL: Pre-competent larva
CL: Competent larva
POL: Post-competent larva
5HT: 5-hydroxytryptamine
CC: Circumoesophageal connectives
LN: Longitudinal nerves
VNC: Ventral nerve cord
WISH: Whole-mount mRNA in situ hybridization
FSW: Filtered sea water
PSU: Practical salinity units
LS: Late-segmented larva
ANOVA: One-way analysis of variance
PBS: Phosphate belanced solution
