# Supplementary material for: Identification of the neuropeptide precursor genes potentially involved in the larval settlement in the Echiuran worm Urechis unicinctus
Source: BMC Genomics. 2020 Dec 14;21:892. doi: 10.1186/s12864-020-07312-4 (PMC7737342; doi:10.1186/s12864-020-07312-4)
Supplement: Supplementary file 1 — Additional file 1: Table S1. Specific primers used in this research. [file 12864_2020_7312_MOESM1_ESM.docx]

**Supplementary Fig. S1** Structures of *Urechis unicinctus* pNPs and identified repetitive peptide motifs.

Structures of *U. unicinctus* pro-neuropeptides (pNP) as indicated, with the predicted signal peptide (green), predicted matured peptides (yellow) and predicted cleavage sites (red), cystein residues (pink) and the C-terminal Glycine of the predicted amidated neuropeptides (cyan). Stop codons are indicated by asterisks (*).For identification of conserved peptide motifs we used NpSearch and manual inspection. For generating peptide logos, we generated multiple sequence alignements with DANMAN and clustalx and created peptide motifs using WebLogo.

>FMRFamide

MPGTCQWAYLFLFIMYIIGHTGATTQSITSQVPIIPLCQALQPYLSAQPQGELHPQQPDDEDEEEREVVESLLDGESKHNKITELEHLLRDIYIDSNKAGVELSLTPRVRRGGNYIRFGRSVPDSQEVHSKYLSSFGTAEKRAGNYLRFGRSATRSRATSEALRDALRKRYMRFGKRDGGYIRFGRSLMGYSGDQVDEADDTSGGENNIDKLNQGNYGLPNDESHPLSPKVNDKQEYNVTQESGKRFLRFGRRFYKFDTKAESDLYMKLYPYGQIKRRYMRFGKRQAELPEDTVYNKRFMKFGKKDNANVAEEDKRYMRFGKKDDSAMTEKEKKFMRFGKKAGGDIPDSDKRYMRFGKKSDIAVSDEEKRFMRFGKKDDSEMSDEEKRFMRFGKKSESDIPESDKRYMRFGKKSDNAMSDEEKRFMRFGKKDGSEMSDEEKRFMRFGKKSTSDIPESNKRYMRFGKKSESDIPDSDKRYMRFGKKSDSEMPDNEDKRFMRFGKKDGSEMSEEENRFVTFGKKSSSDTLEDIKRFMRFGKKSDNERTDERKRFTLYGKPLEGGKRYMRFGKRNSGETLEGDKRFLRFGKKDEEDLSEEGKRFMRFGKKDVGALAGEDKVVENEDDKSLTLFGKKDTRELSDSSKRLLRLGKRDIYSKVSTAL*


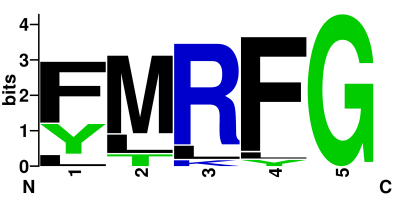


Peptide logo generated from 22 peptides of the FMRFamide peptide pNP.

>MIP/allatostatin-B

MMPSGWRDLTLPILTLILITNSLSHLVTSQEIVPQDGVKEDKRSSTAENPSLLADPSLMVEDGDDVADVDKRKWGSNSMRVWGKRTSEDPLEELEKRKWGDKSMRVWGKRDSEDVDKRKWGSNAMRVWGKRGDGEADKRRWGSNSMRVWGKRSGEEGLDDMDKRKWGSNSMRVWGKRSEEPIGDMEKRKWGYNQMRVWGKRSEDEQEADDVEKRKWGANTMRVWGKRSGEVGDDLMDEEEKRKWGSNTMRVWGKRDSAGDEFGESSLEDNTDTKRKWGSGNMRVWGKRSGEQNTLADVEMEDPLDKRAWGSKSMRVWGKRSISKRSIPDDLELLNDLTDNESRDKRPWGSHNMRVWGKRRSREQGPKRNWQSNLMRVWGKRSSDEDEELEMSKRRWGDNVMRVWGKRAPVDKRAWATGVQRVWGKRGESSGDIEVLDNLLQPSYSETSDDLSAEDMINLLAYLHARLSQSSIEED*


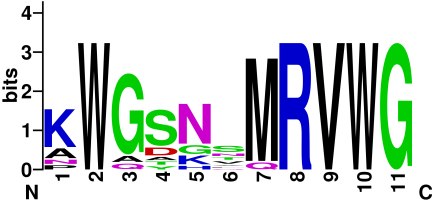


Peptide logo generated from 14 peptides of the MIP peptide pNP.

>WI

MRTNRQNYAVWIVVCAATLGTVTVAALTDATRCSSEKKTEGVALTVLLLGHGCDSPLPHTNLSLASHNDHADEPSSILCHAESDEQGYIRLTVAKTELNDLSLCSALDACVSLSEVEPAPSTAEVDMGWLCVEDEPQHRRLRRDLAHELRAVWSAPSPSGMDWPKRSWSNADTEWLKKRSDIGRWRLNSPYDSNKRWNAAPIEWLKRSSPSYGNNVYDELDGEIYDDLHDDDFNEEKRTWYNSNTDWLKKRGWTNADTEWLKKRSWNDNDMPWLKRWAGNSEVMQWLKKRAWDTSSGLNWLKRNPEIRRGWSNANTEWIKRDLDGTPIHHIGKRSVDDDKQVVEVISEEEAKSAIPDTLSLVKRDWNSAGSDWLKQAEEAKRKWVNSGISWIKRDDHETDKRKWGNSGLSWIKRDHPDEEKRKWGNSGLSWIKRGYPEDEKRKWANSGLSWIKRGDPEEEKRKWGKSGLSWIKRNEPEEEKRKWGNSGLTWIKRDNPEEEKRKWGNSGLSWIKRDDHEDEKRKWANSGLSWIKRADPEEEKRKWGNSGLGWIKRDDPEEEKRKWGNSGLSWIKRDDPEEEKRKWANSGLSWIKRGEHDEGKRKWGNSGLSWIKRKWGNSGLSWIKRNPMEVKRKWGNSGLNWIKRDISDFATEEQVPLELGQQKRNLTPNAAQSKQSQGRSGDKPSPKANTKHH*


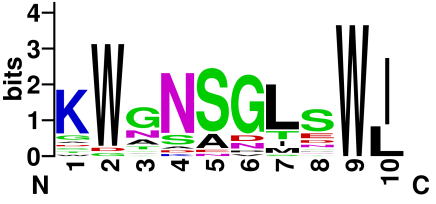


Peptide logo generated from 22 peptides of the WI peptide pNP.

>IRP-1

MANQKRTPSHAILLLCLSAQLIGTLHASERRYRLCGGQLSSVMASVCREHGYNGGRKRAGSALEERRLSDFGLIWQPGSKSYGIRSRQRRGITDECCHQACSMSTLQSYCRVPHEWSPTELEAIVDQMIAPGLVYNRTAIDTSLTPAPRGSVPKTHQERFVTHNSRNRDRFFVTTARRLSSNFYAGEEE*

>IRP-2

MLTGCVRWSLALWVVTMVISGVMAGLERHCSPEDRRKPRGICGRSLADTLHLLCQGIYHKRSSGMDLVPSPPELTNDKSVFMSKTDALSYLQHKRSYSHPLDVGIVCECCVHVCTPRELYQYCASDTGLFGKRSYHAGFPVKTAQKATPGRMFGNNRNSDMGRKPNKVGGKYPTWHANGFEGNDALSHGDAERLFGDDSTEHGGHVESFPNRHRVPHIHYKLEPSRDE*

>IRP-4

MEFSPGCLVVGLLAVFVLLAVGPQGSQADRRRCTVEDYRAGAPKEGFICGNRIPEVLFIICEGSYAGSTSKRAVGKRSEGSGRSWADDNWSDLGLVQEHRESNNLAWDEDDIFMPKKAALSLLRHKRDQFRSGVYCECCRHKCEFREVRHYCSTADRDKRSAPALSMDDPHSEQQKSQETYNKLNLS*

>IRP-5

MYTVSNLNLFSYEFSDETSQCDTVMVFCSSDMIVNQWIRRMDISRCWMSVIVLLVSSGHQQQVLAGRRRCTLDDYRAGAPKSGSICGSRIPEVLSLICNGIFLSPGKKSVDDLAKSGAEWDSPVFIPRQSASSFLRSKRNIAGSQFRTGIYCECCLHPCNIIEMSQYCGQPGERDKRSPLLTAAHQP*

>bursicon-A1

MPRFLVTMQTYIFLVIMTLTQGQTFVPEVSPSSDLQESCKVRRIIHRVDYQGCLPRRMVSLACQGTCRSYTQVSAGQQHVRLERHCSCCQEVQTVTRNATIRCRNDRPYRGRPFKTVVLRLTLPVRCMCRPCSAGVGIVPLELAGIDDKRNLWTAKWMK*

>bursicon-A2

MTISVIAILVSTSLTLCVGFVTSSAYNGDALLHVHTRSVPQCEVGAILLHVRPPSKFAHRCNSTQVLTFGCQGQCSSYSEVNRDDPLRLQHTCSCCEPSKFGVHLAEMNCDGGYKLRAPLKFAMRCSCRPCSSASMDIDMLRRMLDAERLSSTRTAKR*

>bursicon-B

MSRPLYACALNLVKVTVDSRVWQALYSFPDSKRNAHAAGRTSYVTDWSPCRLAMMGRLWSPGNTLQCLFTNLSTAHARTVTCDRNRNVV*

>glycoprotein hormone alpha-2

MMTGSAGPMGSLRLTMTSFMLLCIICIHASNGPAWSTAGCHRLGHTREIHIPNCVKFRVTTNACRGYCTSYAFPSPSWVTDVNPNHQVTSRGECCSITSTHDVHVNVRCTTGIERLTFKSAASCNCDVCRNE*

> glycoprotein beta

MGGTEDKQAEINLRKGGERIRAASCHRSQIADGVTRVATMAQVSLVIACAVSYLVTLTLAEVDVTGTLSCHQRTYQYQISKPFINDLGEVIPCSGLVSVRSCWGRCDSSEVADFRVPYKISQHNVCTYTSRTSRRVRLQDCHADHPDPYAEVFDASDCQCRKCTTANTSCEHITG*

>glycoprotein hormone beta-5-like

MAAWGTRLIVCQLLCLAMTALFMIHEADAFCAIHSYTYKVTRDYLNTSTGQVIECWGSVTVRVCAGRCETGEIADYRAPFKISSHQVCRYAGESTRMVRLQHCPPDHPDPYTFVIDARACECSTCNSAHTYCTTHSYMPPR*

>conopressin/vasotocin-neurophysin

MSGGQFPVYFLIFFVSLPLGSACFIRNCPIGGKRSVNAMSADIGSHHMCMRCGPGGLGQCVGPDICCGAVIGCFIATEESAVCQQENDTPVPCEVGGAPCGSDRQSRCVADGVCCNDASCTLDSDCEDNAGHTSYQDKSDSSVASILPHDLLHYVRQLMASRSFRSRR*

>NPF-1

MDSNCWYRFILVAMAAVLLLATCGSSQDLHGPPERPKVFRNPDELRDYLKALNEYFAIVGRPRFGKRMMAFKRAEAPSFGEFDVNGNGRVDTDEYDGFAFGFRK*

>NPF-2

MATHSSAMSLTIVVYLATVCVLMLACDASYSDDYDRDNTRIAALSHIEPPRRPAQFRNVDELNQYLAELRQYYSILGRPRFGRSVERALGSMTESQQSRRLNHLS*

>NPF-3

MQTIVLIGLVAMTTVATCASFKLYDLPSQQYRLYRTTRTEERGAPKRPQSFQSVAELNRYLSDITDYYTVLGRPRFGKRSLESEKVSRPLSFESKSQLKQALAGAKTSSR*


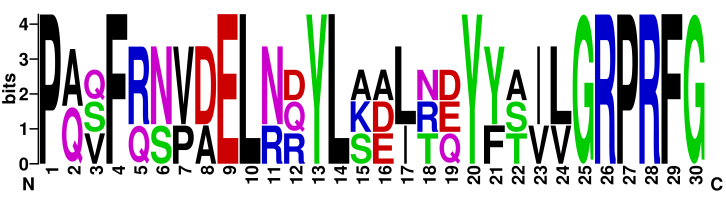


Peptide logo generated from the C-termini of potential peptides yielded by the three *U. unicinctus* NPF pNPs.

>cerebrin

MSGSCVLTILACMACVVVCQVYGGTYENQISAKDRQDILHYAAKILKLALRGDDFGFPSSLQPETKRTSGMLDAVINMPDLYKAGRK*

>AKH

MQRFSTTFIYACAVMALLNIHLVESRFNFMVPGRWGNGAPGKRNTADTSYPSQMLKGAGECDRVNLDTVYVIYRAIQDEAARLARCQQQSSDDLDTPAPLMH*

>7B2

MHAAVVTVLVGVLCACSAQDSLESRLYEDMLMQDLYKRLALLEDTYYPDELASPQGYAPEDLVPPPQYAPDDLAPPQRYSPDAADKSAYSEGISLDSRGDGEAAIRDSEYIEHGPNGGNKGFIHMSGGAGEGKQHLTPEGTQENRQEVKSDEGLPYYCHPPNPCPKGYSPTNDECLDSSEFKDTAESQKSWIATMMKEGLCTCDQEHMFDCPKEMNEQDEMAEMEEQDRIDGIFSSLWADQDKINNSFMTQDKRFTVVAKKSPRIKRSIQEHVARMKKRMETDNKKNPYLSGPKLRTMAKKG*

>DH44

MPDVFACNRNLNSRNSVMTATSSMLGLFVCFLLAMTASAYGYDGDLSALEPSDMEPLQSITDSDPDWVSSLLSADKRRPLSVNQALVPLSNLAYGASRNRQNAQVRNFLNSIGKRSDAGDRAQLLELLASALPSNILRDEDFARKRRGMLSIDMPLGTLSSMLQAERRRQFHDRARSAHNALASLGKRSAPDVDEQDSL*

>achatin

MNSPCCPAIVILAICLIVSPHVLFLSAAAARTHRQDHNDATDQLVTSRRANSRDRREFDEDKRIMEHLRSMIGSHRFTGKRSMSAEKRSMTEENRLSGKRGFADKRGFADKRGFADKRGFADKRGFADKRGFADKRGFADKRIPEQLRWKYFGQVPVPERKHYSEDLGFGSDF*

>calcitonin-1

MWGQLAGLVIAVVCLHGQVCSAAEKPNQQELKEALRIFESLGETRKGLNAVKRVVDDIDGDIAGRHELTDLSKKRRGLDILRRILSEMEADLILEQKRTCQFNLGGHCATESAASVADHWHYLNSAMSPGRKRRDTGLYRKLMTGKLFPQDH*

>calcitonin-2

MWGQLAGLVIAVVCLHGQVCSAAEKPNQQELKEALRIFESLGETRKGLNAVKRVVDDIDGDIAGRQKRLSVCDISAGNAGYACYLQTLSSMDQAKSWLGSDLLSPGKRSAPSGMPINQLAPLNNNAKLPNVRSELTDLSKKRRGLDILRRILSEMEADLILEQKRTCQFNLGGHCATESAASVADHWHYLNSAMSPGRKRRDTGLYRKLMTGKLFPQDH*

>DH31

MQSHASTIVFTLTCLVLISSVTRTQALGEGSRRIQSNEVSDHDLMIDVLLELLGTLRGTNPTIKEKRQMGVDAGYGSRYDVVNRLSSKLMAMQQAADWNGPGRK*

>Pedal peptide 1

MEKSVFALLCLACTMPSCLAADEVSNVKRSISENDMRNEVDKNNRRDLDSLGGGHVPLFGRNLDSLGGGHVPLIGRDLDSLGGGQVPLYGRELDSLGGGHVPLVGRQLDTLGGGHVPLYDRDLDTLGGGHVPLYDRDLDTLGGGHVPLYDRDLDTL**Y**DRDLDTLGGGHVPLYDRGLDTLGGGQVPLYGRQLDTLGGGEVPLYGRQLDTLGGAYVPMARDTNEKRTFDSIGLSRFGGFKRAFNPAKYSNSYGALNALKRAFDSIGHSAFGGLTKRSVERSQRRRRSLNASPDKKSFDSISHSAFGGFDKKSFDSIGHSAFGGMDKKSFDSIGH(…)

>Pedal peptide 2

MNRYINCLWLVALATLLANSRGEDEEKRNFDPIGSSLLKKSFDPIGSSLLKRNFDPIGSSLLKRNFDPIGSSLLKRNFDPIGSSLLKRNFDPIGSSLLKRNFDPIGSSLLKRSFDPIGSSLLKRNFDPIGSSLLKRNFDPIGSSLLKRNFDPIGSSLLKRNFDPIGSSLLKRNFDPIGSSLLKRNFDPIGSSLLKRNFDPIGSSLLKRNFDPIGSSLLKRNFDPIGSSLLKRNFDPIGSSLLKRNFDPIGSSLLKRNFDPIGSSLLKRGHASNEEDFENSIIGKHAIKDLENDMIEERRQLDELADGVIGRELNDHPHLNG*


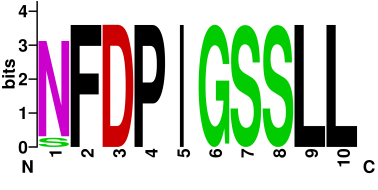


Peptide logo generated from 20 peptides of the pedal peptides from the pedal peptide 2 pNPs.

>allatotropin

MKIGICLLLISVLVSIDAGPHYRNARRSFRIMGASDRFSHGFGKRGDEAVLDNPSDAFSDDITLVTDDELTDILLQNPKLTLAFVQKYLDLNGDGVISKAELTDGSRK*

>sulfakinin

MKIDMRISSSTSLAIIAIIIAICACAVHAAPPPSARDDVVKDDVLLIAKLMAPLQQLSRKLEEIQSYLEHTASDREFNMEMAKRQAWDMDYGYGGGRFGKRTSDDKRYDAFGIAGRFGRSVDHVDPEFNH*

>allatostatin-A

MMSVRWHEVLVGCILVVSFLSASTTASDDDINDVGENEALTFDEDKRAISAGHRYMGLGKRPAINSALRYAGIGKRTMAPSMRYMGLGKRPSLDPAMRFAGLGKRTMDASLKYIGLGKRPMDASMKYIGLGKRVSGETSAEGDSLVGSGLFDTIDQDEAADKRAGMSSAFRFAGLGKRGYSIIQSPGKRSRVGNSMRFMGLGKRDDEDVDDYVPLVDYYQTPGGSDVSEAKRQVARYWSTRRARIDPTFRMMGIGRK*


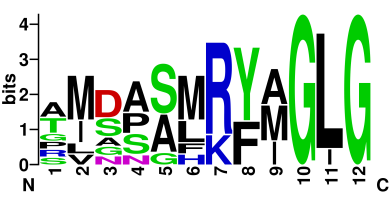


Peptide logo generated from 8 peptides of the allatostatin-A pNP.

>allatostatin-C

MDSKMGLVALVLAVNCLLAVITAASPIEDNFNEIGAKSSRVAFLETKLREDIERELADVMELESQLQKNINMISEKKRQLEIKKREPLHCLVNIVSCWKRK*

>SIFamide

MDSRVMMMSLVTLSLVACLLVNQVTAEPLEDQLPETSGLFFGKRSSHPNMNNLLFGRRSYAQLAAKLQMEEAREFCQTVKEQCARVGLEN*

>leucokinin

MNGPALAVMLTLVHVSGIKSAVVPGQTAHRAMTAATYQDQALFDSFLSKLALAIRPDEPALRPEEPALRSDESALQPVEPALRPGDLSNLMLDIEPSKSASPSTGQEEDDGQAGSEKRSFQPWGGKRASFNPWGGKRQSFNPWGGKRAAFNAWGGKRNSEEYAMKKRPSFNPWGGKRDHISISSQENSESLQEAVEKVRRAFNPWGGKRSLNGWGGQRSSGPTVHEKRQSFNAWGGKRASFNAWGGKRSAAPVDEPSHLLNTLFSPWKEEDSGLHESGL*


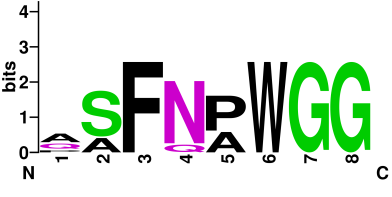


Peptide logo generated from 8 peptides of the leucokinin pNP.

>LFRYamide

MASTYVSLLVVTTLGLMACSESNRRVTRSLSDLDKRLFRYGRNDNIFRYGKRADEDAEAGLLLDNDEDADDHQLAKRLFRWGKRASFFDVDPLESGPVWQGADSSLVDGVVTPLFRHARAGGKITTRTPKQPHVPFRFGDK*


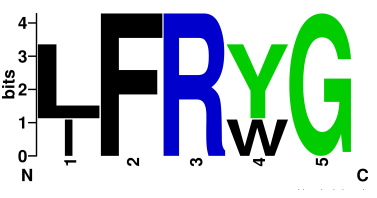


Peptide logo generated from 3 different peptides from the LFRYamide pNP.>prohormone-3

MGGMYLAALVLILTWVQAQSYDIPDWGQFSRRSDCTGGGGRCFSTHECCHSYVCAAYDNYLARRGADDDQDNPEVPGVCVKEKDLRECSSSGDCPSDKACVPLRSTAYHYCVAREHLPPIQKPMYQPLVAGNGRLGTSCSDTAQCMEGLCCQKIRRGRQGTKQMCDRITSISKCLSK*

>myomodulin

MRSLALFAIQFLYVVSSSGEENAHNKREMPMLRMGKRSLREIDVLPPLVPPSYDLAGEDFDERQVWKIPRVGKDLDLPELRLARFPPIPRLGTAYDDILEAYRRDLVDNLDEETMGEMFPLGRHKRSIDDDSEVKNEVKRAAPLPRLGLRDNELDEDERAAPLPRLGMYYRAAPLPRLGFRDLDEEERAAPLPRLGLRDEEEDGYSFDDRAAPLPRLGFRAAPLPRLGFRDVDKKAVNMLRMGRSGVNDGETSEVKRAMSMLRMGKRPMSMLRMGKRPMSMLRMGKRSFEDEVVAKRPMSMLRMGKRSEEEEKRAMSMLRMGKRPMSMLRMGRSEMAYPEDATEEEKRAMGMLRMGRSMGEEQKRAMGMLRMGKRPVSMLRMGKRDVGESQADQNNPEASS*


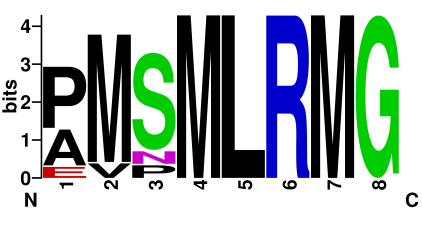


Peptide logo generated from 11 different peptides from the myomodulin pNP.

>whitnin-2

MTSRVSIMDSRSSLLLALLLSALCVLANSLSLPSNDMKESALQMDLDDKETDQGRRATWLETRDLEDDFKELVLLTIQELENEGRLVPGIVAPQQPAKEKRGRWQGFCFKRTRSGRFLPYICWKGDRK*

>FLamide

MVTSKQSSRYGPTTSQPMSICKGLLFVTSIVTVFATPVVSDKDDVSILPHSLTNLDSTSPEVERQRRSSIIDSGSDVWYDEPIDDEKRAKYFLGKRSNRYFLGKRLRNNFLGKRFDSDYESANGGDKRAKYFLGKRDDDSAMDEWQKRAKYFLGKRSEGDLQDEGLNDEENQPLLGKRARYFLGKRESLLADEDYSSLLKRAKYFLGKRAKYFLGKRSSSSPDNYTVPSDLNLLDEKRAKFFLGKKRSDSIDTMDKRQKYFLG*


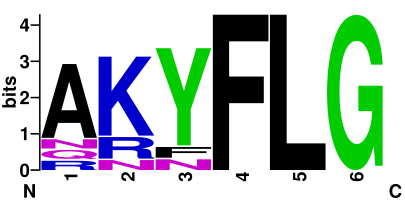


Peptide logo generated from 10 different peptides from the FLamide pNP.

>FVamide

MRHPTLQKVSRSMAVRHIACLALGLSLVHCSAAVDNENSGKIYGENDVGNVDDVKTLLQDVYLGRVLQNKEKQFGAESELNTFVDSQSTERIHDLEKRPRFFVGKRRDLDDDLIKRARFFVGKRNEVSEDLQKKARFFVGKRNEVSEDLQKKARFFVGKRNADNVDEEKISEFFNGKVAEESTKRARFFVGKRNFEDSDEMNKRARFFVGKRNDANDPSMEKKRDRFFVGKRDFDSSLDDIDEIKRARFFVGKRSDNLGKVDEKRARFFVGKRSDEYDEDMDKRARFFVGKRNDEMNEDIEKKPRFFVGKKNRFLGKRPRFFVGKRGYLNDEDEIAKRINDPYYYYSALSHLQNELTKRPRSYVGKRPRFFVGKRSSEESDLDELITEEEKRRHLFVGKRMDSFDDSVEYLEGRSDKRLPRFVGKRDNESKRYRSFVGKRSISPLNRETPSSRLVPGNTGQVDALGH*


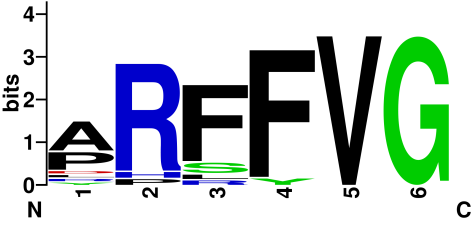


Peptide logo generated from 17 different peptides from the FVamide pNP.

>NKY-1

MYISVCGVSKCQPPHRATMKTQTHQYCNQSAGKMIISLALLAVALVQGSSASPVHRPSSSESVYQLLQEIRDLLHSDMVANQLLRDEALSKNVGNNFDAEGYQPEEENVGHFNQKRNSFWQSMGGPLPVRTRFVSFGSRLEPDQDRSNSASNSMKTMRYGKKR*

>NKY-2

MPDSPTYISDYPTDSGIKPSTDGNQYLAWLHNILSQMRPAPEDQPSTPVKRFSDFSVPYFKYRQDKDKRNDGIWIWMPAQGYVSVPKQQQAIGDEASGKPGKIMRYGK*

>FVRIamide

MGQLSILLPLLLATLSFGDGTLDNICFDICDENSHNLEFEDCQGICRIMLSSPPAYLEDESDGEMFPPVKRSKSSFVRIGRSSRPWWWIRQPVRRKSTFVRIGRSVDGEEEKRASSFVRIGRDPLEQNDGVYPEEEKRPSSFVRIGRPSSFVRIGKKSDDEGYDEKRVSSFVRIGKRPSSFVRIGKSVDHHEEEKRPSSFVRIGKSYDKEEEKRPSSFVRIGKRPSSFVRIGKSSDNMPYEMDKRVSSFVRIGKSDPQSLDETPAKRPSSFVRIGKKDDALVPENEDMDDMKRASAFVRIGKRPSSFVRIGKSSINEPWALAKRPSSFVRIGKSL(…)


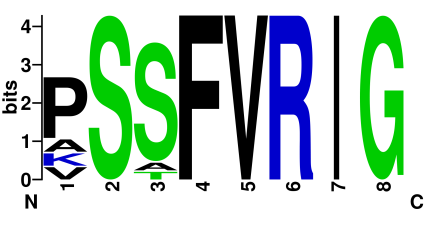


Peptide logo generated from 15 different peptides from the FVRIamide pNP.

>QSGamide

MQPILRTVLLLFFTALIATSISARSLSGYESVRTKRQLGDIYLAAHRADMSLAKQLQPNGCSQIGCGLIDFASSGKKKRSDDYKPYSEEEMRRAQLIHQVLQSLQLTSNDLSDQMSAQ*

>HFAamide

MSQRLMTSPSVRYGLALVLCLLHLTCAFEYSGYCVKMCTWGRGGNLCKCNAVHFAGKRVPQASDVEWATRPVRNLDQGLLGREEVAATSKSDQVDGEFLANSGVRSPNIEADEYMSDVSDVLESLLEEPNTPSGNNDNLEIPILRILAEAMRRRHRTPSGVNVVRQQKQRT*

>RGWamide

MYPTQASLVIVCLWSCLMTVMATDLEDKVNLDAVDKRRGWGKRNGDETDMDKRRGWGKREMTEINEFPSSLDTEEVLESDNEFDKRGWGKRSDAVSLGASGDASWDGDNSGYLLGVEKRRGWGKRSDDGQMDKRRGWGKRSELDLEKRRGWGKRSDFDMEKRRGWGKRGDLDMDKRRGWGKRSFLDSSEMDKRRGWGKRASAAEDTEKRGWGKRSLPLTAALDESDLILEEQPTDVVCSQLKDTLRYYVTKAMQTEVRRQEYCIGSSVA*


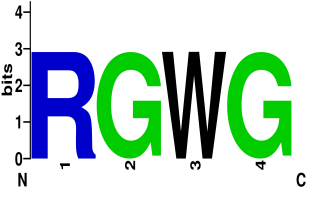


Peptide logo generated from 10 peptides of the RGWamide peptide pNP.>FFamide

MDSRVMMMSLVTLSLVACLLVNQVTAEPLEDQLPETSGLFFGKRSSHPNMNNLLFGRRSYAQLAAKLQMEEAREFCQTVKEQCARVGLEN*

>DLamide

MPRLHPFTISGFLLTCLLCSVHTLTDDDDRSQTPFKRFTAFRSDLGKRSEANSDLENDEEKRFSSFRADLGKRTMDGSDDGVVSLDNMDKRFSAFRADLGKRAGEEDKRFSAFRADLGKRSAGDDDKRFSAFRADLGKRLFDDESDDALTLDDLAKRFSSFRADLGKRSGDDKRFSAFRTDLGKRDDSQVDDAAWDKRFSALRADLGKRFSGLRSDLGKRSGVDEDDDIEEAIKRFSAFRADLGKRAYFRYDLGKRAFSPLDDFEDNAELGMPDNKRAMFRADLGKRTSKSPNELGKRPVYGWHSRFIHPDFGKRLQMFRADLGKRAMFRSDLGKRSAEIPEIQ*


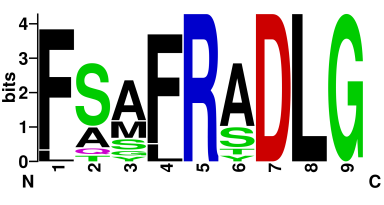


Peptide logo generated from 14 different peptides from the DLamide pNP.

>SLRFamide

MNSFNFLIVVAAIASLHITTAMPRIDSLDANIGDLGLRDAEDDITPRKPTRDNGLGSLLLKNLRFRDLLSQPRPDSNSLYELLQQANLPSSNTDIIYPGELAKRGGVTVSQLAELLHSLKSRDLDDAGVKLQSLRFGRK*

>QERAS

MSRSLSCSSVLLSLVLVSLVSVAMLDTGEAYYLPLAGDSDTLQAIKRDLTQVRTKRYSEYQERASAFCTGMCMYEQRQPYSQCYDYCNWPQNYPKLPTRPRNRRPKTTPPPTTTTTTSTTTTPAPVPLREQSLINDQARLLRMGIPPPKSFLSNPVETPQNDAVAATQSSGKGGRRRKPKKQRKGKNKGGKAKSSTESSGDDSAKECTCRD*

>FXFamide

MASCVKIVSHLVSCNNPTALLFLSLMVITVAYSQPDIQDLSRSWPTKGDEDENLSRAFFDSYADLLHSLAKRTYEYGKLNRRALHLSDYALSGQPAWNKQFMFGKRSVSDDESGNAVFYGEPGANPHLLDNPDKRVKFKFGKRATVDDIDSMNSLMELENVSDEIQKRKKFKFGKRSLEEDLPELMLEGKRLFKFGKKSDESESKRLFKFGKKSVDNFETIENPENKRIFKFGKKSSDGYDEQDAADNKRMFKFGKKSEDGFEIFENPDNKRLFKFGKKSSGDATGMEEPQIKKLFKFGKKSSDNYGDIDISDEKRKFMFGKRSVDQTDSLDEEKRRIFQFGKKSSPYEEDKRAKFMFGKRMDTTDDSYIAEKRPYFQFGKRFDGIDEDKRGPRFTFGKRDEESEYTKRAKFMFGKRRKFMFGKRDEDQMETYSSPVEDSKRMYHPVPYSFTMPGKPMHTSGGMHTMQARPRGSIKTFHFGKRLAESDTEQSTDAQPETGSSSNQAQRR*


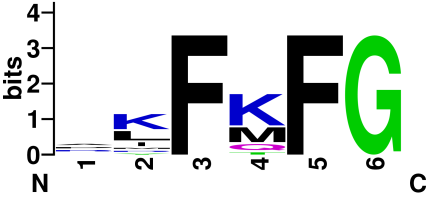


Peptide logo generated from 15 peptides of the FXFamide peptide pNP.

>FILamide

MDTTFRTIYCLFVAGIAVNMSGSILAASNFGPHIVNQPGSRSLRLNDFILGKIKKAYPTYMHFMESRSLPRTPKVSYWDFVMGDANNKRGDLGYINPDYFNLVLGSQPTEYQRRYRRSVDGLQKDVQSTTEKRAHENESPFGTRATIPDYHSFILGSTGGAIPTHRSFRFGAHSNGLPGGQLQFTKRNPQKEVTSGYRNSNEKHSTTKRSTPEYADFVLGKKSMSSNPGFDDFVLGKKSTPQFNEFLLGKKSTPHFDEFILGKKSMPQYDDFILGKKLDPDFNEVFTKEKKSTPTYHDFILGKRAGFPTGRSGYFDFILGKRSQEVTNGSTVNAVK*


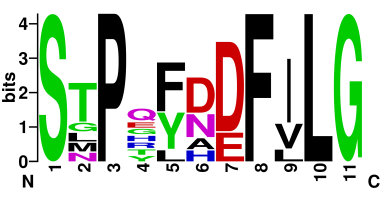


Peptide logo generated from 8 different peptides from the FILamide pNP.

>FW

MEPQPLIFVCLLCIATGAPASDDTNDVIASIWNESNSGDKSRLGKRATEDLSKDSPFWKRAIEDQSKDSAFWKRAMEDQSKDSPFWKRAVENQSKDSPFWKRAIEDQSKDSAFWKRAMEDQSKDSPFWKRAVENQSKDSPFWKRTGDSTDSFWKRGKDEMDGFWKRDDANQASFWKRVPGDEESAFWKKSSGPDTAFWKRNGANDDNFWKRADGTSERFWKRGSEDLETFWKRGGDMSEFWKRAQISPSLNHLLRRLDTNQNEATKKRAPLGEGAGFQKAERRRQQASMLRPQFNPTGW*


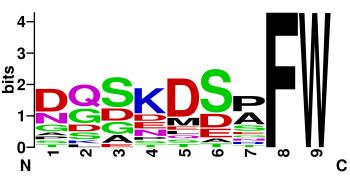


Peptide logo generated from 16 different peptides from the FW pNP.

>FRWamide

MSVLQGVAAPCLVVVLLVAAVTAAAVLPPTLHDTQEAIEQSRDLLCQQLCSLMEEHPGCDCANIDYASNFLSPDTIENDDDDQYIWNDDLEKRANIFRWGKRAAPGILRWGKRAAPGIFRWGKRSSPSSFHWGRDASDFFGGNKRIFRWGKRGSVFKWGKRDDQDASIDDDNSLNMDIKRGKGVFRWGKRSTADSNEDLNLQ*


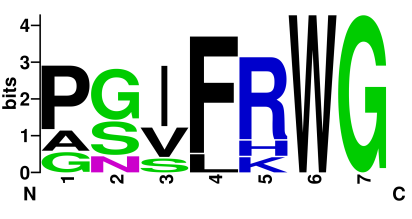


Peptide logo generated from 7 different peptides from the FRWamide pNP.

>ASYY

MEAMFGLRQAALCILAITLVCLSQCVESKFFDDSSFEDRSKRNYIDPACRRCLFDSDDWWSCNSCYAHPGGLIPYFGYSKRSQPKTESDEEAFVSAETEKSQRGVTKRAAFLTCLCCTSLASQECCERCSLASYYGKRNEKRSYETSFVDLGDYGVGGSCSCCNRDPYNFSCCSLHCTKRRRK*
