# Supplementary material for: Identification of the neuropeptide precursor genes potentially involved in the larval settlement in the Echiuran worm Urechis unicinctus
Source: BMC Genomics. 2020 Dec 14;21:892. doi: 10.1186/s12864-020-07312-4 (PMC7737342; doi:10.1186/s12864-020-07312-4)
Supplement: Supplementary file 2 — Additional file 2: Table S2. Detailed information of the identified neuropeptide precursors in U. unicinctus. [file 12864_2020_7312_MOESM2_ESM.pdf]

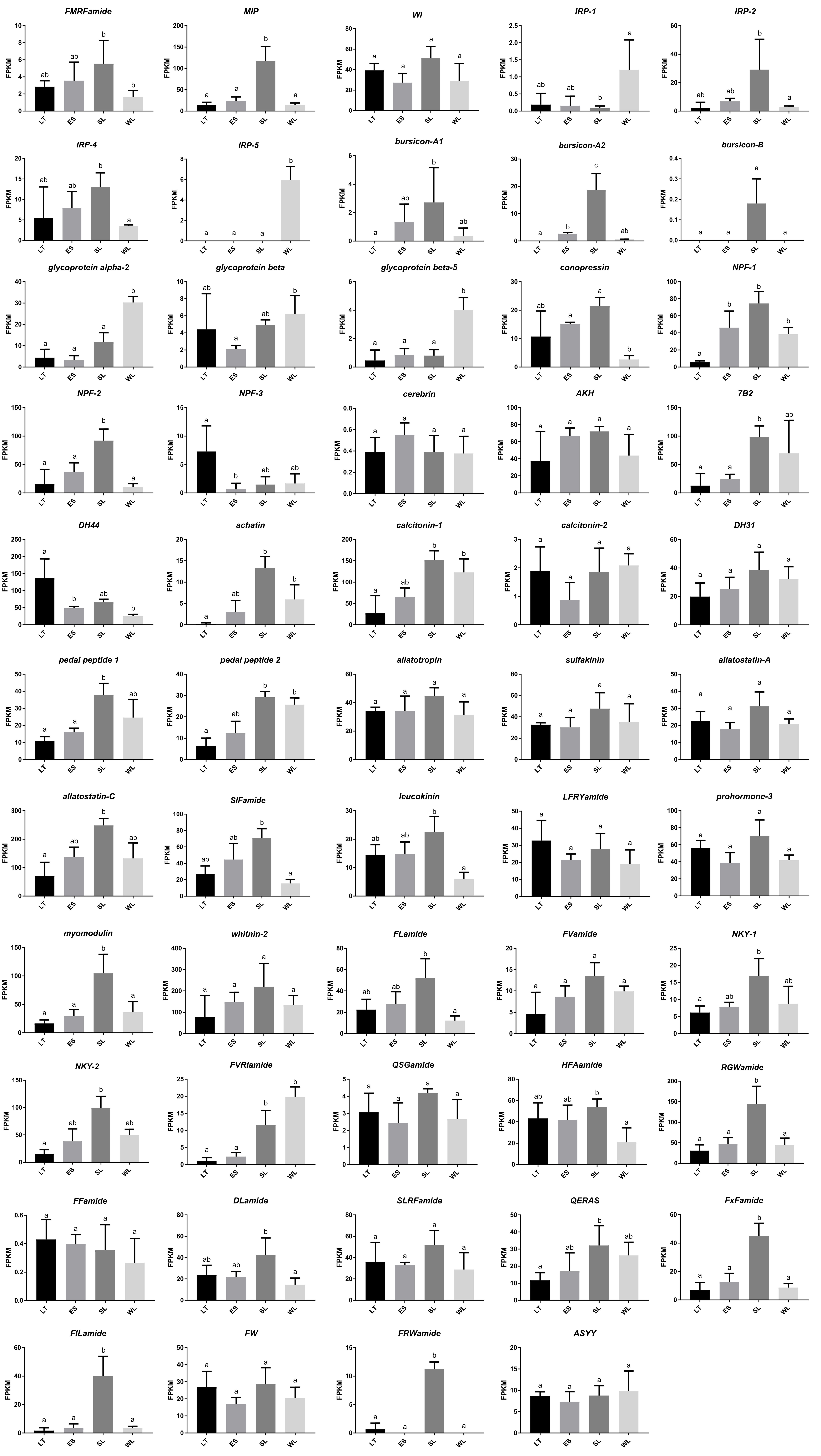

Supplementary Fig. S2 Expression trends of the neuropeptide precursor genes in *U. unicinctus* larval transcriptome.
LT, late-trochophore; ES, early-segmentation larva; SL, segmentation larva; WL, worm-shaped larva. Data are indicated as mean ± SD from triplicate experiments and analyzed using One-way ANOVA followed by Tukey's HSD test. Different letters indicate significant difference between different developmental stages ($p < 0.05$).
