# Supplementary material for: Identification of the neuropeptide precursor genes potentially involved in the larval settlement in the Echiuran worm Urechis unicinctus
Source: BMC Genomics. 2020 Dec 14;21:892. doi: 10.1186/s12864-020-07312-4 (PMC7737342; doi:10.1186/s12864-020-07312-4)
Supplement: Supplementary file 3 — Additional file 3: Figure S1. Structures of U. unicinctus pNPs and identified repetitive peptide motifs. [file 12864_2020_7312_MOESM3_ESM.pdf]

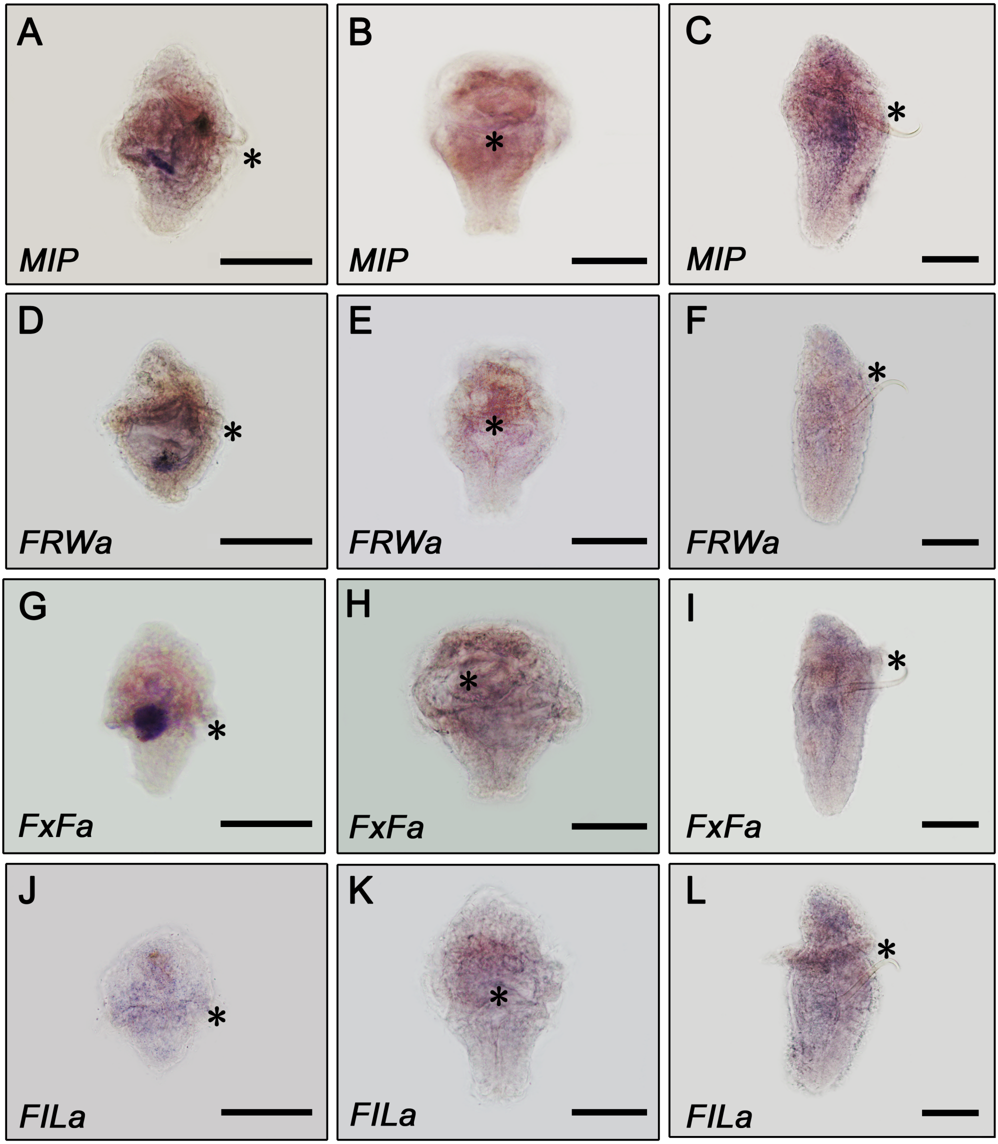

Supplementary Fig. S3 The negative controls of *MIP*, *FRWa*, *FxFa* and *FILa* in *U. unicinctus* larvae detected by whole-mount *in situ* hybridization. (A, D, G and J) correspond to early-trochophore (2 dpf, pelagic larva); (B, E, H and K) correspond to late-trochophore (25 dpf, correspond to LT in transcriptome data, pelagic larva) and (C, F, I and L) correspond to competent larva (35 dpf, correspond to SL in transcriptome data). The asterisk indicates the location of the larvae mouth; B, E, H and K, ventral view; the remaining panels are all lateral views. Scale bars: 200 μm.
